# Brain injury in twin anemia–polycythemia sequence: prevalence, severity and long‐term neurodevelopmental outcome

**DOI:** 10.1002/uog.70209

**Published:** 2026-03-30

**Authors:** M. Rondagh, J. W. van der Spoel, E. Lopriore, J. A. Spekman, L. S. de Vries, J. M. M. van Klink, L. S. A. Tollenaar, F. Slaghekke, S. J. Steggerda, S. G. Groene

**Affiliations:** ^1^ Willem‐Alexander Children's Hospital, Department of Pediatrics, Division of Neonatology Leiden University Medical Center Leiden The Netherlands; ^2^ Fetal Therapy, Department of Obstetrics Leiden University Medical Center Leiden The Netherlands

**Keywords:** brain injury, cranial ultrasound, monochorionic twins, neuroimaging, twin anemia–polycythemia sequence

## Abstract

**Objectives:**

The primary objective of this study was to evaluate the prevalence, type and severity of pre‐ and postnatal brain injury in monochorionic twins with twin anemia–polycythemia sequence (TAPS). Secondary objectives were to conduct within‐pair comparisons between donor and recipient twins of structural cranial ultrasound (cUS) measurements and to evaluate the association between brain injury and neurodevelopmental outcome in TAPS.

**Methods:**

This was a single‐center retrospective cohort study including cases of spontaneous and postlaser TAPS delivered at the Leiden University Medical Center, Leiden, The Netherlands, between May 2002 and December 2024. We evaluated all available pre‐ and postnatal neuroimaging, including ultrasound and magnetic resonance imaging, to identify brain injury. Structural measurements were obtained from cUS performed within 3 days after birth, and were included only if high‐quality images were available for both cotwins. Neurodevelopmental outcome was evaluated using standardized age‐appropriate assessments for cognitive and motor development.

**Results:**

A total of 256 infants were included from 132 pregnancies (124 twin pairs and eight single survivors). Brain injury was observed in 16.8% (43/256) of infants, of which 93.0% (40/43) had a postnatal origin. There was no significant difference in the prevalence of brain injury between cases of spontaneous TAPS (13.5% (20/148)) and postlaser TAPS (21.3% (23/108)) (*P* = 0.1). Severe brain injury was observed in 7.4% (19/256) of the infants, with no significant difference between donor twins (6.5% (8/124)) and recipient twins (8.3% (11/132)) (*P* = 0.54). Lower gestational age at birth was an independent risk factor for brain injury (odds ratio, 1.31 (95% CI, 1.15–1.50); *P* < 0.01). Among infants with moderate‐to‐severe neurodevelopmental impairment (NDI), 57.1% (8/14) had evidence of brain injury. Brain injury alone did not account for the impairment in 71.4% (10/14) of cases with moderate‐to‐severe NDI.

**Conclusions:**

Brain injury was observed in approximately one‐fifth of infants with TAPS, was primarily postnatal in origin and was strongly associated with preterm birth. In the majority of infants with moderate‐to‐severe NDI, the impairment could not be explained by the observed severe brain injury alone, emphasizing its probable multifactorial origin. © 2026 The Author(s). *Ultrasound in Obstetrics & Gynecology* published by John Wiley & Sons Ltd on behalf of International Society of Ultrasound in Obstetrics and Gynecology.

## INTRODUCTION

Monochorionic (MC) twins share a single placenta during pregnancy, which predisposes them to specific complications, such as twin‐to‐twin transfusion syndrome (TTTS), twin anemia–polycythemia sequence (TAPS) and selective fetal growth restriction (sFGR)^1^, ^2^. TAPS is a rare and heterogeneous complication characterized by imbalanced intertwin transfusion through minuscule placental arteriovenous anastomoses, leading to significant intertwin differences in hemoglobin levels without amniotic fluid discordance^1^. TAPS arises spontaneously in 3–5% of MC twin pregnancies, while postlaser TAPS is seen in 1–16% of cases of incomplete coagulation of anastomoses following fetoscopic laser surgery (FLS) for the treatment of TTTS^3^, ^4^. The optimal prenatal management for TAPS remains unclear^5^, ^6^, ^7^.

Previous studies on fetal and neonatal neuroimaging in twins with TAPS have reported varying prevalence rates of brain abnormalities, ranging between 15% and 25%^8^, ^9^. There are several potential mechanisms of brain injury in the case of TAPS, including chronic hypoxia in the donor twin due to anemia, which increases the risk of white‐matter injury; vascular sludging in the recipient twin due to polycythemia, which increases the risk of periventricular/intraventricular hemorrhage and infarction; and brain injury occurring owing to induced preterm birth^8^, ^10^. Brain injury may subsequently result in long‐term neurodevelopmental impairment (NDI). Previous research on long‐term neurodevelopmental outcome after TAPS reported severe NDI in 9% of cases, including cases of both spontaneous and postlaser TAPS^1^, ^11^. The exact cause of NDI in infants with TAPS is still unknown, but it is thought to be multifactorial in origin, including a combination of brain injury, chronic fetal anemia, fetal growth restriction, iron deficiency and hypoalbuminemia^1^, ^12^, ^13^. It is hypothesized that these factors may contribute to impaired brain development and structural deficits. An improved understanding of the mechanisms underlying brain injury and structural brain development in TAPS, and their long‐term consequences, is essential to further improve parental counseling and support the development of preventive strategies such as early intervention. We hypothesize that TAPS is associated with an increased risk of brain injury and altered structural brain development, contributing to adverse long‐term neurodevelopmental outcomes.

The primary aim of this study was to evaluate the prevalence, type and severity of pre‐ and postnatal brain injury in donor and recipient twins with TAPS. Secondary aims were to conduct within‐pair comparisons of structural brain development between donor and recipient twins, as measured on cranial ultrasound (cUS), and to evaluate the association between brain injury and neurodevelopmental outcome in TAPS.

## METHODS

### Study design

This was a retrospective observational cohort study conducted at the Leiden University Medical Center (LUMC), Leiden, The Netherlands, which is the national referral center for complicated MC twin pregnancies and fetal therapy. The study protocol was reviewed and approved by the institutional review board of the LUMC (approval number: 25‐3006). The institutional review board determined that the Medical Research Involving Human Subjects Act was not applicable to this study and informed consent was thereby not required, unless parents had explicitly objected to the use of data for research purposes. All MC twin pregnancies with TAPS diagnosed either pre‐ or postnatally according to the previously published staging systems^14^, ^15^, delivered at LUMC between May 2002 and December 2024, were eligible for inclusion. Prior to 2007, TAPS had not been defined as an official entity, therefore such cases were evaluated retrospectively to determine whether they met the TAPS criteria according to previously published staging systems^14^, ^16^. Currently, a multicenter open‐label randomized controlled trial (RCT) for TAPS is being conducted at LUMC (NL‐OMON26505; https://onderzoekmetmensen.nl/en/trial/46380)^7^. Some of the patients included in the present study are enrolled in the RCT, with the experimental group undergoing FLS and the control group receiving standard care. Patients diagnosed with TAPS within 1 week after FLS for TTTS were excluded from the study, unless TAPS persisted, as fluctuations in fetal middle cerebral artery (MCA) peak systolic velocity (PSV) between twins are common in the immediate postlaser period^17^. Cases complicated by higher‐order multiple pregnancy, congenital or genetic anomaly, termination of pregnancy (TOP) or double intrauterine fetal demise (dIUFD) were excluded, as well as the deceased cotwin after single intrauterine fetal demise (sIUFD) and the deceased cotwin after selective fetal reduction. The surviving cotwin was included in the study cohort if the inclusion criteria were met.

### Prenatal and postnatal characteristics

The following maternal and obstetric characteristics were collected from patient files: maternal age, gestational age (GA) at diagnosis of TAPS, prenatal TAPS stage, donor or recipient twin status, fetal therapy (including FLS and intrauterine transfusion (IUT) with or without partial exchange transfusion (PET)), GA at the time of the prenatal procedure, spontaneous or postlaser TAPS, sFGR (birthweight discordance ≥ 20%), mode of delivery and GA at birth^18^. With regard to fetal therapy, IUT with PET was defined as cases undergoing at least one IUT performed with PET to reduce fetal hematocrit in the recipient twin by replacing a portion of fetal blood with saline solution^19^. The postnatal characteristics collected from patient files included: birth weight, small‐for‐gestational age (SGA) (birth weight < 10^th^ percentile), postnatal TAPS stage, severe neonatal morbidity, neonatal mortality (death within 28 days after birth) and admission to the neonatal intensive care unit (NICU)^20^, ^21^, ^22^. Severe neonatal morbidity was defined as at least one of the following: neonatal sepsis (clinically ill infant and positive blood culture), asphyxia (5‐min Apgar score ≤ 5, need for resuscitation, mechanical ventilation for ≥ 10 min after birth or pH < 7.0, base excess < –16 mmol/L or lactate > 10.0 mmol/L in umbilical cord or venous blood within 1 h after birth), respiratory distress syndrome requiring surfactant or mechanical ventilation, patent ductus arteriosus requiring treatment, necrotizing enterocolitis (NEC) ≥ Stage II, renal failure (plasma creatinine concentration > 130 μmol/L for > 24 h) or retinopathy of prematurity ≥ Stage 3^23^, ^24^, ^25^, ^26^, ^27^. We also reported the use of phototherapy for hyperbilirubinemia, the administration of red blood cell (RBC) transfusions and postnatal PET performed within 28 days after delivery. Administration of RBC transfusion in anemic neonates was based on hemoglobin thresholds that vary with postnatal age and respiratory support status, according to local guidelines. Hemoglobin (in g/dL) and albumin (in g/L) were measured routinely within 48 h after birth. Samples were obtained from the umbilical cord or venous blood.

### Prenatal and postnatal neuroimaging

Fetal ultrasound was routinely performed at the obstetrics department according to the MC twin protocol (every 2 weeks, or more frequently if indicated). Fetal brain magnetic resonance imaging (MRI) was performed at the discretion of the fetal therapy team, primarily if a brain abnormality was observed on fetal ultrasound. If performed, the MRI protocol included T1‐weighted and T2‐weighted sequences or balanced fast field echo sequences, using either a 1.5‐Tesla (T) or a 3.0‐T scanner. When available, susceptibility‐weighted echo planar imaging was also performed. Neonatal cUS was performed routinely in infants with TAPS by an experienced neonatologist within 3 days after birth^28^; resulting structural measurements were only included if high‐quality images were available for both cotwins. Before 2015, neonatal cUS was performed using an Aloka α ultrasound system (Hitachi Medical Systems Holding AG, Zug, Switzerland), while from 2015 onwards a Canon Aplio 400 or Aplio i700 system (Canon Medical Systems Europe BV, Zoetermeer, The Netherlands) was used. Similarly, postnatal brain MRI was performed when deemed necessary by a neonatologist and included three‐dimensional T1‐weighted sequencing and T2‐weighted sequencing in the transverse plane, susceptibility‐weighted imaging and diffusion‐weighted imaging, acquired using a 1.5–3.0‐T MRI system (Achieva, Philips Medical Systems, Best, The Netherlands).

### Brain injury

Brain injury was identified on available pre‐ and postnatal neuroimaging and was confirmed by two experienced neonatologists (L.S.d.V. and S.J.S.). Brain injury was categorized into diffuse or focal, as described by Spruijt *et al*.^29^. Diffuse brain injury included four groups: cystic periventricular leukomalacia (cPVL) ≥ Grade 2 (Group 1); multicystic or generalized encephalomalacia (Group 2); migration or gyration disorders (Group 3); and ventriculomegaly (VM) (atrial width of the lateral ventricle or ventricular index > 97^th^ percentile on pre‐ or postnatal imaging, respectively) and/or severe volume loss (Group 4)^30^, ^31^, ^32^, ^33^. Focal brain injury also included four groups: infarction (Group 5); intraventricular hemorrhage (IVH) with or without post‐hemorrhagic ventricular dilation (PHVD) (Group 6); IVH with periventricular hemorrhagic infarction (PVHI) (Group 7); and cerebellar hemorrhage classified as punctate (< 4 mm), limited (involving < 1/3 of a single cerebellar hemisphere) or extensive (involving > 1/3 of a cerebellar hemisphere or cerebral parenchymal hemorrhage) (Group 8)^34^, ^35^. IVH was classified using the widely adopted grading system: Grade I, germinal matrix hemorrhage; Grade II, IVH without PHVD; Grade III, IVH with PHVD^34^, ^35^. Brain injury confirmed within 24 h after birth was classified as prenatal brain injury. Severe brain injury was defined as at least one of the following: IVH ≥ Grade 3, disorders of cortical migration, PVL ≥ Grade 2, VM, PHVD, arterial or venous infarction, cerebral parenchymal hemorrhage or extensive cerebellar hemorrhage^20^.

### Structural cranial ultrasound measurements

Postnatal cUS measurements were obtained offline using Clinical Assistant (RVC Medical IT B.V., Amersfoort, The Netherlands) by a single researcher (M.R.) with experience in neonatal cUS imaging, who was blinded to clinical characteristics and outcome. Ultrasound measurements were selected based on their reproducibility and high reliability as established in the previous literature^36^, as well as based on their potential association with NDI. Ultrasound measurements were obtained as previously described by Groene *et al*.^20^, other than for biparietal diameter (BPD) and frontal occipital diameter, which were measured from outer‐to‐outer border to ensure higher reliability (Table S1)^37^. All measurements were corrected for BPD to account for individual variability in intracranial size, thereby enhancing the accuracy and validity of between‐subject comparisons. The pericallosal branch of the anterior cerebral artery (ACA) was used for postnatal Doppler assessment as it is easily accessible through the anterior fontanelle and is routinely evaluated in neonatal cUS, whereas MCA Doppler measurements are not consistently obtained postnatally, despite their regular prenatal use in the context of TAPS. Measurements were repeated in a random 10% sample of the population (20 infants) to allow for calculation of the intraclass correlation coefficient (ICC) for every measurement (two‐way mixed‐effects model). ICC values of < 0.50 indicated poor reliability, 0.50–0.75 indicated moderate reliability, > 0.75–0.90 indicated good reliability and > 0.90 indicated excellent reliability^37^. Ultrasound measurements could not be obtained when cUS images were unavailable or of low quality. Measurements were only included when the corresponding parameter could be obtained for both twins.

### Neurodevelopmental outcome

Neurodevelopmental assessment was performed for all infants over 2 years of age as part of previous long‐term follow‐up studies and routine clinical care for infants with TAPS^1^, ^11^, ^38^. Cognitive and motor development were assessed using standardized age‐appropriate tests. The Dutch version of the Bayley Scales of Infant and Toddler Development, third edition (Bayley‐III)^1^, was administered at 2–3 years of corrected age. The Dutch version of the Wechsler Preschool and Primary Scale of Intelligence, third or fourth edition (WPPSI‐III‐NL, WPPSI‐IV‐NL), was used for infants aged 3–6 years, and the Dutch Wechsler Intelligence Scale for Children, third, fourth or fifth edition (WISC‐III‐NL, WISC‐IV‐NL, WISC‐V‐NL) was used for infants ≥ 7 years of age^1^. These tests provide a cognitive index score (for Bayley‐III) or a full‐scale intelligence quotient (FSIQ) (for WPPSI and WISC), corrected for preterm birth, following a score metric with a mean of 100 and a standard deviation of 15. Hearing impairment was defined as mild (hearing loss up to 30 decibels) or severe (bilateral deafness), and visual impairment as mild (requiring treatment by an ophthalmologist, strabismus or a correction of at least ± 3.0 by glasses or contact lenses) or severe (blindness or partially sighted)^24^. Cerebral palsy (CP) was classified using the Gross Motor Function Classification System^39^. Mild NDI was defined as mild cognitive or motor impairment (composite Bayley‐III < 85 and/or FSIQ < 85 − 1 SD), mild hearing or visual impairment or the presence of Grade‐1 CP. Moderate‐to‐severe NDI was defined as severe cognitive or motor impairment (composite Bayley‐III < 70 and/or FSIQ < 70 − 2 SD), severe hearing or visual impairment, postnatal epilepsy or moderate‐to‐severe CP (≥ Grade 2)^1^, ^24^. When there was an inconsistency in neurodevelopmental outcome between findings at 2, 5 and 8 years of age, the outcome assessed at the last visit was deemed definitive.

### Statistical analysis

All statistical analysis was conducted using SPSS version 29.0 (IBM Corp., Armonk, NY, USA). Normally distributed data are presented as mean ± SD, non‐normally distributed data are reported as median (interquartile range (IQR)) and categorical variables are presented as *n* (%). Prenatal characteristics were analyzed at the pregnancy level, whereas all other analyses were performed at the individual level. Categorical variables were compared using the chi‐square test, Fisher's exact test (for unpaired data with an observed count < 10 or expected count < 5) or the McNemar test for paired data. Continuous variables were analyzed using the paired Student's *t*‐test for normally distributed paired data or the Mann–Whitney *U*‐test for unpaired non‐normally distributed data. Comparisons between cotwins were performed using paired analysis or generalized estimating equations (GEE) to account for within‐twin correlation, which were applied for brain injury, neurodevelopmental outcome and cUS measurements. Comparisons between TAPS types (spontaneous or postlaser) were performed using unpaired tests. Potential risk factors for brain injury were analyzed using a binary logistic regression analysis with GEE. Risk factors for brain injury were selected *a priori* based on theoretical relevance and prior evidence associated with altered hemodynamics, hypoxia, placental dysfunction or known contributors to fetal and neonatal brain injury, and included donor status, postlaser TAPS, IUT, FLS for TAPS, GA at birth and SGA^1^, ^4^, ^5^, ^8^, ^9^, ^11^. Univariable linear regression with GEE was used to assess within‐pair comparisons for cUS measurements. Multivariable linear regression with GEE was conducted to adjust for BPD. To control for multiple testing in the analysis of postnatal cUS measurements, a Benjamini–Hochberg false‐discovery rate correction was applied. For these comparisons, both unadjusted *P*‐values and adjusted *P*‐values (q‐values) are reported, with statistically significant results after adjustment indicated. For all other analyses, unadjusted *P*‐values are presented; *P* or q < 0.05 was considered statistically significant^40^.

## RESULTS

### Study population

**Figure 1:**
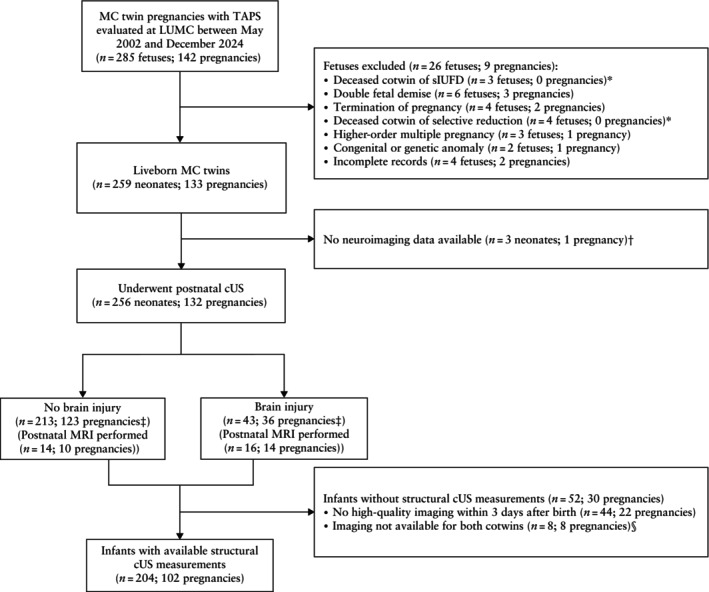
Flowchart summarizing inclusion in study of monochorionic (MC) twin pregnancies with twin anemia–polycythemia sequence (TAPS) evaluated at Leiden University Medical Center (LUMC). *Pregnancies not excluded as surviving cotwin was included. †Includes one twin pair and one cotwin; one pregnancy was not excluded as data were available for other cotwin. ‡Brain‐injury status was assessed at the cotwin level, therefore sum of pregnancies does not equal total number of pregnancies; within a single pregnancy, one cotwin may have brain injury while the other may not. §Exclusion of eight infants corresponds to eight pregnancies, as the cotwin in each pregnancy had been excluded previously. cUS, cranial ultrasound; MRI, magnetic resonance imaging; sIUFD, single intrauterine fetal demise.

In total, 285 fetuses (142 pregnancies) were diagnosed with TAPS pre‐ or postnatally and delivered between May 2002 and December 2024. Of these, 26 fetuses (nine pregnancies) were excluded (Figure 1). Of the remaining 259 infants (133 pregnancies) eligible for inclusion, 256 infants (132 pregnancies; 124 twin pairs and eight single survivors) underwent postnatal cUS and comprised the study cohort.

### Clinical characteristics

Baseline characteristics are presented in Table 1, categorized by the overall population of pregnancies with TAPS and according to spontaneous TAPS or postlaser TAPS. TAPS was diagnosed prenatally in 90.2% (119/132) of pregnancies at a mean ± SD GA of 24.2 ± 4.4 weeks. Overall, IUT was performed in 25.8% (34/132) of pregnancies, and IUT with PET in 9.8% (13/132) of all pregnancies. sFGR was present in 25.8% (34/132) of pregnancies, with a median intertwin birthweight discordance of 31.4% (IQR, 26.3–35.1%). Of pregnancies with spontaneous TAPS, 30.3% (23/76) underwent FLS following inclusion in the ongoing TAPS‐RCT, at a mean GA of 23.2 ± 2.9 weeks. In cases of postlaser TAPS, FLS for TTTS was performed at a mean GA of 21.2 ± 3.6 weeks. Three pregnancies in this group underwent a second FLS procedure for postlaser TAPS. The median GA at birth was lower in pregnancies with postlaser TAPS (31.8 (IQR, 28.9–33.7) weeks) compared to those with spontaneous TAPS (33.1 (IQR, 30.1–34.9) weeks) (*P* = 0.02). Overall, the donor twins had a significantly lower mean birth weight (1566 ± 528 g) compared with recipient twins (1729 ± 527 g) (*P* < 0.01) (Table 2). Care was transitioned from a curative to a palliative approach in 12 cases owing to severe brain injury (*n* = 9), severe respiratory and/or circulatory complications (*n* = 2) and NEC (*n* = 1). The rate of neonatal mortality was higher in donor twins (7.3% (9/124)) compared with recipient twins (2.3% (3/132)) (*P* < 0.01). A total of 50.8% (63/124) of donor twins received a RBC transfusion for anemia, whereas 23.5% (31/132) of recipient twins underwent postnatal PET for polycythemia.

**Table 1:** Prenatal and obstetric characteristics of 132 monochorionic pregnancies (124 twin pairs, 8 single survivors) with twin anemia–polycythemia sequence (TAPS), overall and according to spontaneous or postlaser TAPS

Characteristic	Overall TAPS (*n* = 132 pregnancies)	Spontaneous TAPS (*n* = 76 pregnancies)	Postlaser TAPS (*n* = 56 pregnancies)
Maternal age (years)	31 ± 5	31 ± 5	31 ± 5
Prenatal TAPS diagnosis	119 (90.2)	68 (89.5)	51 (91.1)
GA at prenatal diagnosis (weeks)	24.2 ± 4.4	24.2 ± 4.9	24.3 ± 3.7
Prenatal TAPS stage			
Stage 1	23/119 (19.3)	13/68 (19.1)	10/51 (19.6)
Stage 2	66/119 (55.5)	41/68 (60.3)	25/51 (49.0)
Stage 3	23/119 (19.3)	12/68 (17.6)	11/51 (21.6)
Stage 4	5/119 (4.2)	2/68 (2.9)	3/51 (5.9)
Stage 5	2/119 (1.7)	0/68 (0)	2/51 (3.9)
Fetal therapy			
Expectant management	39 (29.5)	39 (51.3)	0 (0)
IUT without PET	21 (15.9)	12 (15.8)	9 (16.1)
IUT with PET	13 (9.8)	6 (7.9)	7 (12.5)
FLS	79 (59.8)	23 (30.3)	56 (100)
Cesarean section	74 (56.1)	44 (57.9)	30 (53.6)
GA at birth (weeks)	32.4 (29.7–34.6)	33.1 (30.1–34.9)	31.8 (28.9–33.7)
Postnatal TAPS stage			
Stage 1	21/73 (28.8)	7/33 (21.2)	14/40 (35.0)
Stage 2	22/73 (30.1)	10/33 (30.3)	12/40 (30.0)
Stage 3	19/73 (26.0)	7/33 (21.2)	12/40 (30.0)
Stage 4	8/73 (11.0)	6/33 (18.2)	2/40 (5.0)
Stage 5	3/73 (4.1)	3/33 (9.1)	0/40 (0)

Data are given as mean ± SD, *n* (%), *n*/*N* (%) or median (interquartile range). FLS, fetoscopic laser surgery; GA, gestational age; IUT, intrauterine transfusion; PET, partial exchange transfusion.

**Table 2:** Postnatal characteristic of 256 neonates with previous twin anemia–polycythemia sequence (TAPS), overall and according to spontaneous or postlaser TAPS, stratified by donor and recipient twin

	All TAPS twins (*n* = 256 neonates)		Spontaneous TAPS (*n* = 148 neonates)		Postlaser TAPS (*n* = 108 neonates)	
Characteristic	Donor (*n* = 124)	Recipient (*n* = 132)	Donor (*n* = 72)	Recipient (*n* = 76)	Donor (*n* = 52)	Recipient (*n* = 56)
Birth weight (g)	1566 ± 528	1729 ± 527	1636 ± 557	1860 ± 543	1470 ± 473	1550 ± 451
SGA	65 (52.4)	35 (26.5)	43 (59.7)	17 (22.4)	22 (42.3)	18 (32.1)
Hemoglobin (g/dL)	11.9 ± 4.7	21.3 ± 3.2	12.3 ± 5.2	21.0 ± 3.3	11.4 ± 3.8	21.6 ± 2.9
Albumin (g/L)	24.7 ± 7.3	32.3 ± 4.8	23.6 ± 7.6	32.5 ± 5.1	25.8 ± 6.8	32.1 ± 4.5
RBC transfusion	63 (50.8)	8 (6.1)	34 (47.2)	4 (5.3)	29 (55.8)	4 (7.1)
Postnatal PET	0 (0)	31 (23.5)	0 (0)	11 (14.5)	0 (0)	20 (35.7)
Phototherapy	55 (44.4)	95 (72.0)	27 (37.5)	51 (67.1)	28 (53.8)	44 (78.6)
NICU admission	116 (93.5)	124 (93.9)	67 (93.1)	70 (92.1)	49 (94.2)	54 (96.4)
Severe neonatal morbidity	47 (37.9)	57 (43.2)	23 (31.9)	31 (40.8)	24 (46.2)	26 (46.4)
Neonatal mortality	9 (7.3)	3 (2.3)	2 (2.8)	1 (1.3)	7 (13.5)	2 (3.6)

Data are given as mean ± SD or *n* (%). NICU, neonatal intensive care unit; PET, partial exchange transfusion; RBC, red blood cell; SGA, small‐for‐gestational age.

### Brain injury

Brain injury was observed in 16.8% (43/256) of twins, with a prenatal origin in 7.0% (3/43) (Table 3, Figure S1). Twins with postlaser TAPS showed no significant difference in the prevalence of brain injury compared to twins with spontaneous TAPS (21.3% (23/108) *vs* 13.5% (20/148)) (*P* = 0.1). We observed no difference in the prevalence of brain injury in donor (20.2% (25/124)) compared with recipient twins (13.6% (18/132)) (*P* = 0.12). Overall, the most frequently observed brain injury was IVH (Groups 6 and 7; 65.1% (28/43)). Among the infants with IVH, three (10.7%) had PVHI and six (21.4%) developed PHVD.

**Table 3:** Brain injury in 256 infants with twin anemia–polycythemia sequence (TAPS), overall and according to spontaneous or postlaser TAPS, stratified by donor and recipient twin

	All TAPS twins (*n* = 256 infants)			Spontaneous TAPS (*n* = 148 infants)			Postlaser TAPS (*n* = 108 infants)		
Outcome	Donor (*n* = 124)	Recipient (*n* = 132)	*P*	Donor (*n* = 72)	Recipient (*n* = 76)	*P*	Donor (*n* = 52)	Recipient (*n* = 56)	*P*
All brain injury	25 (20.2)	18 (13.6)	0.12	9 (12.5)	11 (14.5)	0.71	16 (30.8)	7 (12.5)	0.01
Postnatal	23/25 (92.0)	17/18 (94.4)	0.75	9/9 (100)	10/11 (90.9)	1.00	14/16 (87.5)	7/7 (100)	1.00
Severe	8 (6.5)	11 (8.3)	0.54	1 (1.4)	8 (10.5)	0.02	7 (13.5)	3 (5.4)	0.14
Diffuse brain injury	6 (4.8)	2 (1.5)	0.15	0 (0)	1 (1.3)	1.00	6 (11.5)	1 (1.8)	0.08
Group 1	4 (3.2)	2 (1.5)	0.38	0 (0)	1 (1.3)	1.00	4 (7.7)	1 (1.8)	0.19
cPVL Grade 2	1/4 (25.0)	2/2 (100)	0.40	—	1/1 (100)	N/A	1/4 (25.0)	1/1 (100)	0.40
cPVL Grade 3	3/4 (75.0)	0/2 (0)	0.40	—	0/1 (0)	N/A	3/4 (75.0)	0/1 (0)	0.40
Group 2	0 (0)	0 (0)	N/A	0 (0)	0 (0)	N/A	0 (0)	0 (0)	N/A
Group 3	1 (0.8)	0 (0)	0.48	0 (0)	0 (0)	N/A	1 (1.9)	0 (0)	0.48
Group 4	1 (0.8)	0 (0)	0.48	0 (0)	0 (0)	N/A	1 (1.9)	0 (0)	0.48
Focal brain injury	19 (15.3)	16 (12.1)	0.39	9 (12.5)	10 (13.2)	0.92	10 (19.2)	6 (10.7)	0.17
Group 5	0 (0)	3 (2.3)	0.25	0 (0)	2 (2.6)	0.50	0 (0)	1 (1.8)	1.00
Group 6	15 (12.1)	10 (7.6)	0.15	6 (8.3)	6 (7.9)	0.90	9 (17.3)	4 (7.1)	0.05
IVH Grade I	9/15 (60.0)	3/10 (30.0)	0.13	4/6 (66.7)	2/6 (33.3)	0.48	5/9 (55.6)	1/4 (25.0)	0.31
IVH Grade II	4/15 (26.7)	3/10 (30.0)	0.77	2/6 (33.3)	1/6 (16.7)	0.48	2/9 (22.2)	2/4 (50.0)	0.17
IVH Grade III	2/15 (13.3)	4/10 (40.0)	0.41	0 (0)	3/6 (50.0)	0.18	2/9 (22.2)	1/4 (25.0)	0.68
Group 7	1 (0.8)	2 (1.5)	0.61	1 (1.4)	1 (1.3)	0.97	0 (0)	1 (1.8)	1.00
Group 8	3 (2.4)	1 (0.8)	0.31	2 (2.8)	1 (1.3)	0.54	1 (1.9)	0 (0)	0.48

Data are given as *n* (%) or *n*/*N* (%). Group 1, cystic periventricular leukomalacia (cPVL) ≥ Grade 2; Group 2, multicystic or generalized encephalomalacia; Group 3, migration or gyration disorders; Group 4, ventriculomegaly (atrial width of the lateral ventricle or ventricular index > 97^th^ percentile) and/or severe volume loss; Group 5, infarction; Group 6, intraventricular hemorrhage (IVH) with or without post‐hemorrhagic ventricular dilation (PHVD); Group 7, IVH with periventricular hemorrhagic infarction; Group 8, cerebellar hemorrhage. IVH Grade I, germinal matrix hemorrhage; IVH Grade II, IVH without PHVD; IVH Grade III, IVH with PHVD. N/A, not applicable.

Severe brain injury was observed in 7.4% (19/256) of twins, with no significant difference between donor (6.5% (8/124)) and recipient twins (8.3% (11/132)) (*P* = 0.54), or between spontaneous TAPS (6.1% (9/148)) and postlaser TAPS (9.3% (10/108)) (*P* = 0.35). In spontaneous TAPS, only 1.4% (1/72) of donor twins and 10.5% (8/76) of recipient twins were observed to have severe brain injury (*P* = 0.02), while in postlaser TAPS it was observed in 13.5% (7/52) of donor twins and 5.4% (3/56) of recipient twins (*P* = 0.14). No significant differences were observed for overall (*P* = 0.44) or severe (*P* = 0.14) brain injury between spontaneous TAPS cases treated with FLS and those not treated with FLS.

Of those with a known outcome, most infants with severe brain injury (93.3% (14/15)) experienced an adverse clinical outcome, including neonatal death, CP ≥ Grade 2, CP ≥ Grade 2 with epilepsy, mild cognitive impairment and mild motor impairment. One infant had a normal outcome, and outcomes were unknown for four cases (Table S2).

There were 28 surviving infants with brain injury and long‐term follow‐up. NDI was observed in 60.7% (17/28) of infants, including 32.1% (9/28) with mild NDI and 28.6% (8/28) with moderate‐to‐severe NDI. Of the seven surviving infants with severe brain injury and long‐term follow‐up, 71.4% (5/7) had NDI, including 14.3% (1/7) with mild NDI and 57.1% (4/7) with moderate‐to‐severe NDI.

In prenatal counseling, understanding the prevalence of brain injury and severe brain injury is important and cases of deceased cotwins after sIUFD, dIUFD, deceased cotwins after selective reduction and TOP should be included, rather than focusing solely on liveborn twins with TAPS. To ensure that the prevalence of brain injury was not affected by the excluded cases, the calculations were repeated including those cases. The overall prevalence of brain injury and severe brain injury were unchanged.

### Fetal and neonatal MRI findings

Prenatal MRI was performed in 3.5% (9/256) of fetuses owing to abnormalities observed during fetal ultrasound. Only 11.1% (1/9) of fetal MRI scans showed severe brain injury. Postnatal MRI was performed in 11.7% (30/256) of infants, with 53.3% (16/30) showing brain injury, including 26.7% (8/30) with severe brain injury. Indications for postnatal MRI included suspected white‐matter injury on cUS (*n* = 13), extreme prematurity (*n* = 8), suspected deep gray‐matter injury (*n* = 3), postnatal epilepsy (*n* = 3), postnatal cytomegalovirus infection (*n* = 2) and suspected sinovenous thrombosis on cUS (*n* = 1).

### Risk factors for brain injury

Univariate logistic regression analysis was performed to evaluate potential risk factors for brain injury, including donor status, TAPS type (spontaneous or postlaser), IUT, GA at birth, SGA and FLS for TAPS (Table 4). Only lower GA at birth and IUT showed a significant association with increased odds of brain injury (odds ratio (OR), 1.31 (95% CI, 1.15–1.50) and 2.45 (95% CI, 1.11–5.41), respectively). Similarly, lower GA at birth was associated with increased odds of severe brain injury (OR, 1.37 (95% CI, 1.10–1.71)).

**Table 4:** Potential risk factors for brain injury in 256 infants with twin anemia–polycythemia sequence (TAPS)

			Univariate analysis	
Risk factor	Brain injury (*n* = 43)	No brain injury (*n* = 213)	OR (95% CI)	*P*
Donor twin	25 (58.1)	99 (46.5)	1.60 (0.88–2.89)	0.12
Postlaser TAPS	23 (53.5)	85 (39.9)	1.71 (0.83–3.50)	0.14
IUT*	9 (20.9)	24 (11.3)	2.45 (1.11–5.41)	0.03
FLS for TAPS†	9 (20.9)	41 (19.2)	1.19 (0.50–2.83)	0.69
GA at birth	29.7 (27.7–31.9)	33.0 (30.1–34.7)	1.31 (1.15–1.50)‡	< 0.01
SGA	17 (39.5)	83 (39.0)	1.07 (0.55–2.08)	0.85

Data are given as *n* (%) or median (interquartile range).

* Donor twin in one pregnancy that underwent intrauterine transfusion (IUT) was excluded from analysis due to single intrauterine fetal demise.

† Includes fetoscopic laser surgery (FLS) in cases of spontaneous TAPS and second FLS for TAPS in cases of postlaser TAPS; in two pregnancies that underwent FLS, only one cotwin was included in the analysis.

‡ Per 1‐week reduction in gestational age (GA). OR, odds ratio; SGA, small‐for‐gestational age.

### Cranial ultrasound measurements

Postnatal cUS imaging for structural measurements was available for both twins in 102 pregnancies (204 infants), performed at a median of 1 (IQR, 0–1) day of age (Table 5). The ICC showed excellent reliability for all cUS measurements (Table S1). Overall, Doppler cUS showed significantly lower PSV in the ACA of recipient twins compared with donor twins (q < 0.01). In the spontaneous TAPS group, donor twins had a significantly smaller basal ganglia insula width (q < 0.01), BPD (q < 0.01), left and right deep gray‐matter height (q < 0.01 for both) and transverse cerebellar diameter (q < 0.05) compared with recipient twins. After correction for BPD, none of the cUS measurements remained significantly different.

**Table 5:** Postnatal cranial ultrasound measurements in 204 neonates with previous twin anemia–polycythemia sequence (TAPS), overall and according to spontaneous or postlaser TAPS, stratified by donor and recipient twin

	All TAPS twins (*n* = 204 neonates)				Spontaneous TAPS (*n* = 136 neonates)				Postlaser TAPS (*n* = 68 neonates)			
Measurement	Donor (*n* = 102)	Recipient (*n* = 102)	*P*	q‐value	Donor (*n* = 68)	Recipient (*n* = 68)	*P*	q‐value	Donor (*n* = 34)	Recipient (*n* = 34)	*P*	q‐value
Doppler measurements												
ACA‐PSV (cm/s)	21.1 (15.9–25.4)	17.4 (13.3–21.5)	< 0.01	< 0.01	21.3 (15.9–25.4)	18.0 (14.9–22.1)	0.02	0.05	20.1 (16.4–23.4)	13.0 (10.4–17.9)	0.01	0.07
ACA‐EDV (cm/s)	5.1 (3.6–6.7)	4.1 (2.7–5.5)	0.04	0.07	5.0 (3.7–6.7)	4.5 (3.0–5.7)	0.27	0.45	5.5 (3.6–6.5)	3.0 (2.1–4.2)	0.01	0.07
ACA‐RI	0.8 (0.7–0.8)	0.8 (0.7–0.8)	0.79	0.91	0.7 (0.7–0.8)	0.7 (0.6–0.8)	0.86	0.92	0.8 (0.7–0.8)	0.8 (0.8–0.8)	0.16	0.29
Structural measurements												
Left VI (mm)	9.5 (8.7–10.5)	9.7 (8.8–10.6)	0.72	0.90	9.7 (8.4–10.5)	9.4 (8.7–10.7)	0.67	0.84	9.5 (9.0–10.3)	10.0 (9.0–10.4)	0.18	0.31
Right VI (mm)	9.7 (8.6–10.9)	9.7 (8.7–10.5)	0.86	0.92	9.6 (8.5–10.8)	9.8 (8.6–10.8)	0.48	0.65	10.2 (9.0–11.5)	9.7 (8.9–10.2)	0.55	0.64
BGIW (mm)	23.8 (21.2–25.7)	24.1 (22.5–26.1)	< 0.01	< 0.01	24.2 (21.5–26.1)	24.5 (23.0–26.4)	< 0.01	< 0.01	22.9 (21.2–25.0)	22.9 (21.0–25.2)	0.08	0.23
BPD (mm)	75.0 (69.9–80.8)	76.4 (71.8–81.5)	< 0.01	< 0.01	75.6 (70.1–81.6	78.5 (73.5–81.9)	< 0.01	< 0.01	72.8 (69.7–76.4)	73.4 (69.4–76.0)	0.54	0.64
FOD (mm)	98.2 (80.8–102.7)	99.1 (81.8–103.3)	0.99	0.99	99.5 (93.5–107.5)	100.9 (91.6–103.8)	0.92	0.92	77.8 (74.9–80.8)	77.2 (76.9–77.4)	0.52	0.64
ICH (mm)	71.8 (67.7–77.0)	73.7 (67.5–77.9)	0.16	0.22	73.5 (69.1–77.1)	74.4 (68.7–79.0)	0.06	0.13	69.0 (65.1–74.1)	69.9 (65.9–74.6)	1.00	1.00
CC (mm)	40.0 (36.9–42.3)	40.7 (37.8–43.1)	0.04	0.07	40.6 (37.2–42.9)	41.8 (38.1–43.5)	0.12	0.22	38.3 (36.7–41.1)	38.4 (37.3–41.3)	0.16	0.29
CCF (mm)	44.2 (40.8–46.5)	44.2 (42.5–46.6)	0.10	0.16	44.9 (41.2–47.1)	45.0 (43.2–47.5)	0.45	0.65	42.7 (39.6–44.8)	43.6 (40.7–44.4)	0.10	0.26
VH (mm)	18.8 (17.3–20.8)	19.6 (17.9–20.9)	0.05	0.08	19.1 (17.5–21.1)	19.6 (17.8–21.1)	0.75	0.86	17.9 (16.0–20.1)	19.2 (18.0–20.4)	< 0.01	0.02
Left DGMH (mm)	17.8 (16.5–19.3)	18.5 (17.5–20.1)	< 0.01	< 0.01	18.1 (16.7–19.6)	18.9 (17.8–20.1)	< 0.01	< 0.01	17.2 (15.5–18.7)	18.3 (16.7–19.5)	0.08	0.23
Right DGMH (mm)	18.3 (16.8–19.3)	18.8 (17.9–19.9)	< 0.01	< 0.01	18.5 (16.8–19.6)	19.3 (18.3–20.5)	< 0.01	< 0.01	18.0 (16.6–19.0)	18.4 (17.1–19.3)	0.83	0.89
TCD (mm)	39.8 (34.6–44.0)	39.4 (35.4–45.0)	0.02	0.04	42.0 (35.1–44.9)	41.6 (36.0–46.0)	0.02	< 0.05	35.7 (28.3–38.8)	36.3 (31.5–37.7)	0.50	0.64

Data are given as median (interquartile range). To control for multiple testing, the false‐discovery rate was maintained at 5% using the Benjamini–Hochberg (BH) procedure. Both unadjusted (*P*) and BH‐monotone‐adjusted (q) values are reported. ACA, anterior cerebral artery; BGIW, basal ganglia insular width; BPD, biparietal diameter; CC, corpus callosal length; CCF, corpus callosum–fastigium length; DGMH, deep gray‐matter height; EDV, end‐diastolic velocity; FOD, fronto‐occipital diameter; ICH, intracranial height; PSV, peak systolic velocity; RI, resistance index; TCD, transverse cerebellar diameter; VH, vermian height; VI, ventricular index.

### Neurodevelopmental impairment

Neurodevelopmental follow‐up data were available for 64.8% (166/256) of infants, recorded at a median age of 29 (IQR, 25–69) months (Table 6). For the infants without follow‐up data, 41.1% (37/90) had not yet reached 2 years of age or had died in the neonatal period, 43.3% (39/90) were lost to follow‐up and 15.6% (14/90) had no formal indication for follow‐up according to the current national NICU follow‐up guideline^41^.

**Table 6:** Neurodevelopmental outcome of 256 infants with previous twin anemia–polycythemia sequence (TAPS), overall and according to spontaneous or postlaser TAPS, stratified by donor and recipient twin

	All TAPS (*n* = 256 infants)			Spontaneous TAPS (*n* = 148 infants)			Postlaser TAPS (*n* = 108 infants)		
Outcome	Donor (*n* = 124)	Recipient (*n* = 132)	*P*	Donor (*n* = 72)	Recipient (*n* = 76)	*P*	Donor (*n* = 52)	Recipient (*n* = 56)	*P*
Known neurodevelopmental outcome	81 (65.3)	85 (64.4)	0.88	48 (66.7)	49 (64.5)	0.78	33 (63.5)	36 (64.3)	0.93
Normal	52/81 (64.2)	70/85 (82.4)	< 0.01	32/48 (66.7)	42/49 (85.7)	0.01	20/33 (60.6)	28/36 (77.8)	0.18
NDI	29/81 (35.8)	15/85 (17.6)	< 0.01	16/48 (33.3)	7/49 (14.3)	0.01	13/33 (39.4)	8/36 (22.2)	0.09
Mild	21/81 (25.9)	9/85 (10.6)	< 0.01	11/48 (22.9)	5/49 (10.2)	0.06	10/33 (30.3)	4/36 (11.1)	0.03
Moderate‐to‐severe	8/81 (9.9)	6/85 (7.1)	0.72	5/48 (10.4)	2/49 (4.1)	0.26	3/33 (9.1)	4/36 (11.1)	0.40
Unknown neurodevelopmental outcome	43 (34.7)	47 (35.6)	0.95	24 (33.3)	27 (35.5)	0.94	19 (36.5)	20 (35.7)	0.97
Neonatal death	10/43 (23.3)	3/47 (6.4)	< 0.01	2/24 (8.3)	1/27 (3.7)	0.471	8/19 (42.1)	2/20 (10.0)	< 0.01
< 2 years of age at time of follow‐up	12/43 (27.9)	12/47 (25.5)	0.30	10/24 (41.7)	10/27 (37.0)	0.20	2/19 (10.5)	2/20 (10.0)	0.76
No indication for follow‐up	6/43 (14.0)	8/47 (17.0)	0.28	6/24 (25.0)	8/27 (29.6)	0.28	0/19 (0)	0/20 (0)	N/A
Lost to follow‐up	15/43 (34.9)	24/47 (51.1)	0.01	6/24 (25.0)	8/27 (29.6)	0.32	9/19 (47.4)	16/20 (80.0)	< 0.01

Data are given as *n* (%) or *n*/*N* (%). N/A, not applicable; NDI, neurodevelopmental impairment.

Among those with follow‐up data, 73.5% (122/166) had normal neurodevelopment, while 18.1% (30/166) had mild NDI and 8.4% (14/166) had moderate‐to‐severe NDI. In infants with normal neurodevelopment, 9.0% (11/122) showed evidence of brain injury on neuroimaging, including 1.6% (2/122) with severe brain injury (bilateral Grade‐3 IVH with PHVD and caudate nucleus infarction). Among those with mild NDI, 30.0% (9/30) showed evidence of brain injury on neuroimaging, including 3.3% (1/30) with severe brain injury (cPVL Grade 2). Of infants with moderate‐to‐severe NDI, 57.1% (8/14) showed evidence of brain injury on neuroimaging, with half of these cases (28.6% (4/14)) demonstrating severe brain injury (Table S3). Moderate‐to‐severe NDI included bilateral deafness (*n* = 4; all donor twins with spontaneous TAPS), postnatal epilepsy (*n* = 3), CP ≥ Grade 2 (*n* = 2), CP Grade 2 with postnatal epilepsy (*n* = 1), severe cognitive impairment (*n* = 3) and severe motor impairment (*n* = 1). Brain injury alone did not account for the impairment in 71.4% (10/14) of cases of moderate‐to‐severe NDI. This included four cases of bilateral deafness, three of severe cognitive impairment, one of severe motor impairment and two of epilepsy.

## DISCUSSION

This retrospective study of 256 liveborn twins with TAPS found evidence of brain injury in 17%, with 93% of cases of brain injury thought to occur postnatally. The prevalence of brain injury and severe brain injury did not differ between twins with spontaneous TAPS and those with postlaser TAPS. Brain injury was primarily associated with preterm birth, with 31% higher odds of brain injury per each week of earlier birth, and most commonly presented as IVH. Over 90% of the infants with severe brain injury experienced an adverse clinical outcome, predominately neonatal death or CP ≥ Grade 2.

The majority of twins with moderate‐to‐severe NDI presented with bilateral deafness or severe cognitive impairment. In some cases, the absence of brain injury detected on ultrasound did not account for the severity of the observed NDI, suggesting a multifactorial origin. However, the limited sensitivity of ultrasound to detect subtle abnormalities, including global white‐matter injury and altered brain maturation, gyrification and connectivity, raises concerns regarding its adequacy to fully capture the scope of such impairments. Advanced MRI techniques are needed to assess brain maturation, gyrification and connectivity to better understand the unexplained NDI. In the spontaneous‐TAPS group, postnatal cUS measurements showed significantly smaller BPD, deep gray‐matter height and transverse cerebellar diameter in donor twins compared with recipient twins. However, after correction for BPD, these findings did not remain significant.

Previous MRI studies in pregnancies with TAPS reported brain abnormalities in 15–25% of cases, primarily of prenatal origin^8^, ^9^. Tricca *et al*.^8^ observed a brain abnormality in 21.8% of fetuses, which was more frequent in donor twins (75%) than recipient twins (25%). However, this might be an overestimation, as only those with suspected brain abnormalities were included. Most findings, such as brain swelling and venous prominence, resolved postnatally, suggesting that these findings are of limited clinical relevance. Rosen *et al*.^9^ also excluded cases of fetal demise and reported a 15% prevalence of brain injury, predominantly observed in recipient twins. In contrast, our study found no difference in the prevalence of brain injury between donor and recipient twins. The higher prevalence of prenatal brain injury in previous MRI studies compared with ours may be due to the limited use of fetal MRI at our center (performed in 4% of fetuses), the focus on brain injury rather than resolved abnormalities and different selection criteria, as we excluded deceased cotwins after sIUFD in our analysis. Since previous MRI studies on TAPS did not report neurodevelopmental outcomes, the true significance of the observed abnormalities remains unclear, limiting our understanding of their impact on long‐term motor and cognitive function.

In our study, a lower GA at birth was identified as an independent risk factor for brain injury, consistent with findings from previous studies^29^, ^42^. Comparison with GA‐matched preterm cohorts suggests that the prevalence of brain injury related to extreme preterm birth in cases of TAPS is similar to that observed in our study, although differences in imaging modalities and definitions limit comparability^43^, ^44^. FLS carries an inherent risk of preterm delivery, with mean GA at birth reported to be around 32–34 weeks after FLS, which may also partly account for the lower GA at birth in this group^11^, ^15^. To date, there is no consensus on the optimal timing of delivery and management strategy in both spontaneous and postlaser TAPS, and the decision should carefully balance the risks of *in‐utero* distress and potential complications related to preterm birth. The ongoing multicenter TAPS‐RCT is evaluating whether FLS improves outcome, focusing on GA at birth as the primary outcome. Despite the potential risks of FLS, the additional burden of FLS is considered low compared with the natural course of TAPS^7^. In our study there was no difference in both overall and severe brain injury between spontaneous TAPS cases treated with FLS compared with those not treated with FLS. However, this finding should be interpreted with caution, as the retrospective study design and the small sample size resulted in the study not being powered adequately to evaluate this outcome.

While donor twins with spontaneous TAPS are at a lower risk for severe brain injury than recipient twins, we observed an increased prevalence of NDI related to bilateral deafness in donor twins^1^. Several factors may contribute to this observation. First, chronic fetal anemia may result in prolonged hypoxia, affecting both brain and, hypothetically, auditory nerve development^45^. Second, both SGA, affecting 52% of donor twins in our cohort, and hypoglycemia, present in 53% of donor twins in a different cohort, have been strongly associated with NDI in MC twin pregnancies^24^, ^45^. Finally, previous studies have shown an association between hypoalbuminemia and iron deficiency with reduced intracranial volume or adverse long‐term outcome^13^, ^46^, ^47^.

Given the emerging evidence for the high risk of brain injury in both spontaneous and postlaser TAPS cases^8^, ^9^, standardized prenatal and postnatal brain imaging protocols are needed, and may improve the detection rate of brain injury and enhance parent counseling regarding long‐term neurodevelopmental outcomes. Fetal MRI should be considered when ultrasound findings are inconclusive or when abnormality is suspected. Our study showed a high risk of brain injury, which was predominantly of postnatal origin, in twins with TAPS. We recommend performing postnatal cUS within 72 h after birth for all MC twin pregnancies complicated by TAPS, irrespective of GA at birth, to detect potential brain injury, in line with our local protocol. In cases of mild white‐matter abnormality, follow‐up ultrasound may help to monitor progression. For persistent or severe abnormalities, early MRI should be considered to guide further management. Furthermore, routine long‐term follow‐up and hearing screening should be performed in all cases of TAPS^1^, ^11^.

### Strengths and limitations

To our knowledge, this is the largest study to date on the prevalence, type and severity of brain injury in TAPS, and the first study to evaluate structural cUS measurements in both spontaneous and postlaser TAPS. We adopted a systematic, strictly defined approach to examining brain‐injury patterns^29^, ^48^. We also evaluated neurodevelopmental outcome and its relation to the observed brain injury, which is frequently underreported in studies on brain injury in TAPS^8^, ^9^.

This study also has some limitations. First, the retrospective design was subject to missing data and potential selection bias, which could impact on the generalizability of our findings. Second, fetal MRI was sporadically performed within our population, which may lead to an underestimation of prenatal brain injury or the inability to visualize transient lesions. Third, reliance on cUS, with few postnatal MRI scans performed, may have limited the detection of subtle or diffuse white‐matter injury and accounted for some discordance between the evidence of brain injury and the development of NDI. Variation in ultrasound quality prevented assessment of whether findings such as periventricular echogenicity were transient or progressive. Fourth, over the study period there have been advances in fetal and neonatal neuroimaging, improvements in FLS (since the start of the TAPS trial) and significant enhancements in postnatal care (e.g. improved awareness in our referral centers). These changes may have influenced the observed prevalence of brain injury. Lastly, correction of cUS measurements is ideally performed using head circumference, for better accuracy. However, owing to limited MRI use and missing head‐circumference data, BPD was chosen as an alternative, given its strong correlation with total brain volume^49^.

### Conclusions

Twins with TAPS, including both spontaneous and postlaser TAPS, are at an increased risk of both pre‐ and postnatal brain injury and subsequent NDI. This risk increases with earlier GA at delivery. In the majority of twins with moderate‐to‐severe NDI, the finding of postnatal brain injury could not fully explain the observed development of NDI, primarily among donor twins, which suggests that NDI has a multifactorial origin. This study highlights the importance of routine perinatal neuroimaging, using ultrasound and MRI, and long‐term follow‐up in cases of TAPS.

## Supporting information


**Table S1** Description of cranial ultrasound measurements, including intraclass correlation coefficients.
**Table S2** Clinical characteristics, imaging findings and outcome of twins with twin anemia–polycythemia sequence (TAPS) with severe brain injury.
**Table S3** Infants with previous anemia–polycythemia sequence (TAPS) with moderate‐to‐severe neurodevelopmental impairment.

**Figure S1** Magnetic resonance imaging (MRI) of brain injury in twins with twin anemia–polycythemia sequence (TAPS).

## Data Availability

The data that support the findings of this study are available on request from the corresponding author. The data are not publicly available due to privacy or ethical restrictions.
