# Supplementary material for: Brain injury in twin anemia–polycythemia sequence: prevalence, severity and long‐term neurodevelopmental outcome
Source: Ultrasound Obstet Gynecol. 2026 Mar 30;67(4):470–81. doi: 10.1002/uog.70209 (PMC13040136; doi:10.1002/uog.70209)
Supplement: Supplementary file 1 — Table S1 Description of cranial ultrasound measurements, including intraclass correlation coefficients. Table S2 Clinical characteristics, imaging findings and outcome of twins with twin anemia–polycythemia sequence (TAPS) with severe brain injury. Table S3 Infants with previous anemia–polycythemia sequence (TAPS) with moderate‐to‐severe neurodevelopmental impairment. [file UOG-67-470-s001.docx]

**Table S1. Description of cranial ultrasound measurements, including intraclass correlation coefficients.***Intra-rater reliability was assessed using the intraclass correlation coefficient (ICC), based on a two-way mixed-effects model, consistency type, and single measures (ICC[3,1]). ICC values and 95% confidence intervals were calculated from ANOVA-derived mean squares, with subjects as random effects and measurement sessions as fixed effects.*

| Plane and fontanelle | Measurement | Abbreviation | ICC | Reliability |
| --- | --- | --- | --- | --- |
| Coronal, anterior fontanelle | Ventricular index | VI | Right: 0.95 (95% CI, 0.86-0.98) | Excellent |
|  |  |  | Left: 0.92 (95% CI, 0.81-0.97) | Excellent |
|  | Basal ganglia insula width | BGIW | 0.98 (95% CI, 0.96-0.99) | Excellent |
|  | Biparietal diameter | BPD | 0.99 (95% CI, 0.99-1.00) | Excellent |
| Sagittal, anterior fontanelle | Fronto-occipital diameter | FOD | 0.98 (95% CI, 0.95-1.00) | Excellent |
|  | Intracranial height | ICH | 0.99 (95% CI, 0.99-1.00) | Excellent |
|  | Corpus callosal length | CC | 0.99 (95% CI, 0.99-1.00) | Excellent |
|  | Corpus callosum-fastigium length | CCF | 0.99 (95% CI, 0.99-1.00) | Excellent |
|  | Vermis height | VH | 0.96 (95% CI, 0.87-0.99) | Excellent |
| Parasagittal, anterior fontanelle | Deep gray matter height,  left and right | DGMH | Right: 0.94 (95% CI, 0.85-0.98) | Excellent |
|  |  |  | Left: 0.96 (95% CI, 0.88-0.99) | Excellent |
| Coronal, mastoid fontanelle | Transverse cerebellar diameter | TCD | 0.99 (95% CI, 0.99-1.00) | Excellent |

**Table S2** **Clinical characteristics, imaging findings and outcome of twins with twin anemia–polycythemia sequence (TAPS) with severe brain injury.**

| Case | Donor or recipient | TAPS type | GA at TAPS diagnosis (weeks, days) | Fetal therapy (GA at treatment, weeks) | GA at birth (weeks, days) | BW (gram, percentile) | Neonatal co-morbidity | Origin of brain injury | Imaging | Imaging findings | Outcome (age at follow-up, months) |
| --- | --- | --- | --- | --- | --- | --- | --- | --- | --- | --- | --- |
| #1 | Donor | Spontaneous TAPS | 22+4 | FLS (23)  IUT (25) | 24+6 | 660 (p22) | RDS  NEC (IIIB) | Postnatal | cUS | Bilateral IVH grade 3 with PVHI | Neonatal death |
| #2 | Donor | Postlaser TAPS | 20+2 | FLS (20) | 25+0 | 640 (p<10) | Sepsis  RDS  PDA | Postnatal | cUS | Bilateral IVH grade 3 with PHVD | Neonatal death |
| #3 | Donor | Postlaser TAPS | 20+6 | FLS (21) | 28+1 | 1122 (p24) | RDS | Postnatal | cUS | cPVL grade 2  Unilateral IVH grade 1 | Neonatal death |
| #4 | Donor | Postlaser TAPS | 27+3 | FLS (2) | 27+5 | 930 (p11) | Sepsis  RDS | Postnatal | cUS | IVH grade 3 unilateral and grade 2 unilateral  PVL grade 1 | Neonatal death |
| #5 | Donor | Postlaser TAPS | Postnatal | FLS (21) | 26+2 | 1165 (p97) | RDS | Prenatal | US  cUS  MRI | Severe microcephaly, bilateral polymicrogyria of parietal lobes and partly frontal and occipital lobes, and agenesis of the corpus callosum. | Neonatal death |
| #6 | Donor | Postlaser TAPS | 24+2 | FLS (18) | 30+1 | 1693 (p79) | RDS  PDA | Postnatal | cUS | cPVL grade 3 | Neonatal death |
| #7 | Donor | Postlaser TAPS | NR | FLS (24, 26) | 32+4 | 1653 (p<10) | - | Postnatal | cUS | cPVL grade 3 | CP grade 3 |
| #8 | Donor | Postlaser TAPS | 23+4 | FLS (22)  IUT with PET (23)  IUT (25, 26, 27) | 27+5 | 980 (p10) | RDS | Postnatal | cUS  MRI | cPVL grade 3  Unilateral IVH grade 2  Unilateral punctate (<4mm) cerebellar hemorrhages | Neonatal death |
| #9 | Recipient | Spontaneous  TAPS | Postnatal | Expectant | 30+1 | 1534 (p48) | Sepsis  RDS | Postnatal | cUS  MRI | Bilateral IVH grade 3 with PHVD  PWML | WPPSI-III-NL FSIQ: 101, motor: NR (60m) |
| #10 | Recipient | Spontaneous  TAPS | 28+4 | IUT (31) | 31+5 | 1790 (p41) | RDS | Postnatal | cUS | Bilateral IVH grade 2 with PHVD | Lost to follow-up |
| #11 | Recipient | Spontaneous  TAPS | 25+1 | Expectant | 29+4 | 1257 (p23) | - | Postnatal | cUS  MRI | Caudate Nucleus infarction | Bayley-III composite cognitive score: 124, motor: 115 WPPSI-IV-NL FSIQ: 111 (69m) |
| #12 | Recipient | Spontaneous  TAPS | 22+4 | FLS (23)  IUT (25) | 24+6 | 680 (p30) | RDS  NEC (IIIB) | Postnatal | cUS | Bilateral IVH grade 3 with PHVD | Lost to follow-up |
| #13 | Recipient | Spontaneous TAPS | 25+5 | FLS (26) | 32+3 | 1775 (p29) | RDS | Postnatal | cUS | cPVL grade 2 | Bayley-III composite cognitive score: 115, motor: 81 (25m) |
| #14 | Recipient | Spontaneous TAPS | 20+6 | Expectant | 36+3 | 2920 (p56) | - | Prenatal | cUS  MRI | Unilateral temporal infarction | Postnatal epilepsy Bayley-III composite cognitive score: 81, motor: 101 (24m) |
| #15 | Recipient | Spontaneous TAPS | 24+6 | FLS (26) | 26+6 | 1070 (p80) | Sepsis  RDS | Postnatal | cUS  MRI | Bilateral IVH grade 3 with PHVD  Punctate (<4mm) cerebellar hemorrhages | Neonatal death |
| #16 | Recipient | Spontaneous TAPS | 29+4 | Expectant | 29+5 | 1140 (p<10) | RDS | Postnatal | cUS  MRI | Caudate Nucleus infarction Unilateral IVH grade 2 | Below 2 years of age |
| #17 | Recipient | Postlaser TAPS | 20+2 | FLS (20) | 25 | 820 (p69) | RDS | Postnatal | cUS | Bilateral IVH grade 3 with PHVD | Neonatal death |
| #18 | Recipient | Postlaser TAPS | 28 | FLS (22) | 31+5 | 1555 (p17) | RDS | Postnatal | cUS MRI | cPVL grade 2 | CP grade 2 |
| #19 | Recipient | Postlaser TAPS | 28 | FLS (27) | 28+2 | 971 (p<10) | Sepsis | Postnatal | cUS  MRI | MCA stroke | CP grade 2  Postnatal epilepsy |

*Abbreviations: Bayley-III, Bayley Scales of Infant and Toddler Development, third edition; BW, birthweight; BWD, birthweight discordance; CC, corpus callosal length; CP, cerebral palsy; cPVL, cystic periventricular leukomalacia; cUS, cranial ultrasound; FLS, fetoscopic laser surgery; FSIQ, full scale intelligent quotient; GA, gestational age; IUT, intrauterine transfusion; IVH, intraventricular hemorrhage; MCA, middle cerebral artery; MRI, magnetic resonance imaging; N/A, not applicable; NEC, necrotizing enterocolitis; NR, not reported; PDA, patent ductus arteriosus; PHVD, post-hemorrhagic ventricle dilation; PVHI, periventricular hemorrhagic infarction, PVWML, periventricular white matter lesions; RDS, respiratory distress syndrome; sFGR, selective fetal growth restriction; SGA, small for gestational age; TAPS, twin anemia polycythemia sequence; US, ultrasound; WPPSI-III-NL, Wechsler Preschool and Primary Scale of Intelligence, third edition; WPPSI-IV-NL: Wechsler Preschool and Primary Scale of Intelligence, fourth edition.*

**Table S3. Infants with previous anemia–polycythemia sequence (TAPS) with moderate-to-severe neurodevelopmental impairment***Abbreviations: BW, birthweight;* *Bayley 3: Bayley Scales of Infant and Toddler Development, third edition; CP, cerebral palsy; cPVL, cystic periventricular leukomalacia; FLS, fetoscopic laser surgery; FSIQ: full-scale intelligent quotient; GA, gestational age; Hb, hemoglobin; IUT, intrauterine transfusion; IVH, intraventricular hemorrhage; MCA, middle cerebral artery; NR, not reported; PET, partial exchange transfusion; RBC, red blood cell; RDS, respiratory distress syndrome; TAPS, twin anemia polycythemia sequence; WES, whole exosome sequencing; WISC-V-NL, Wechsler Intelligence Scale for Children, fifth edition; WPPSI-III-NL, Wechsler Preschool and Primary Scale of Intelligence, third edition. # Case number of Table 4. ** *Brain injury alone did not account for the moderate-to-severe neurodevelopmental impairment*

| Case | Donor or recipient | TAPS type | GA at TAPS diagnosis (weeks, days) | Fetal therapy  (GA at treatment, weeks) | GA at birth (weeks, days) | BW (gram, percentile) | Neonatal morbidity | Hb at birth (g/dL, treatment) | Brain injury | Long-term outcome (age at follow-up, months) |
| --- | --- | --- | --- | --- | --- | --- | --- | --- | --- | --- |
| #1* | Donor | spontaneous TAPS | NR | Expectant | 30+1 | 843  (p<1) | RDS | 3.1 (4x RBC transfusion) | Punctate cerebellar hemorrhages | Bilateral deafness  WPPSI-III-NL FSIQ: 80 (60m) |
| #2* | Donor | spontaneous TAPS | 25+6 | Expectant | 28+6 | 1250 (p29) | RDS | 8.1 (1x RBC transfusion) | - | Bilateral deafness  WISC-V-NL FSIQ: 89 (94m) |
| #3* | Donor | spontaneous TAPS | 28+3 | FLS (28) | 28+4 | 1176 (p37) | RDS | 4.1 (3x RBC transfusion) | - | Bilateral deafness  WISC-V-NL FSIQ: 104 (102m) |
| #4* | Donor | spontaneous TAPS | 22+1 | Expectant | 34+3 | 1955  (p<10) | - | 15.5 | - | Bayley composite cognitive score: 87, motor: 69 (26m) |
| #5* | Donor | spontaneous TAPS | 28+0 | IUT (29, 31) | 33+3 | 1700 (p<10) | Asphyxia  RDS | 4.8 (3x RBC transfusion) | IVH grade 2 | Bilateral deafness  Bayley composite cognitive score: 105, motor: 112 (26m) |
| #6* | Donor | post-laser TAPS | 26+2 | FLS (23) | 29+1 | 1079 (p<5) | Sepsis  RDS  Renal failure | 15.8 (6x RBC transfusion) | Unilateral IVH grade 1 | Bayley composite cognitive score: 69, motor: 83 (24m) |
| #7 | Donor | post-laser TAPS | NR | FLS (24, 26)  IUT (26) | 32+4 | 1653 (p<10) | - | 13.5 | cPVL grade 3 (#7) | CP grade 3 |
| #8* | Donor | post-laser TAPS | 24+2 | FLS (22)  IUT (28, 28, 30) | 30+1 | 1370 (p16) | - | 12.4 | Unilateral limited cerebellar hemorrhage | Postnatal epilepsy (triple WES negative) Bayley composite cognitive score: 77, motor: 89 (24m) |
| #9* | Recipient | spontaneous TAPS | 22+5 | Expectant | 27+0 | 880 (p<10) | Sepsis  RDS | 24.6 | - | Bayley composite cognitive score: 63, motor: NR (25m) |
| #10 | Recipient | spontaneous TAPS | 20+6 | Expectant | 36+3 | 2920 (p56) | - | 25.4 | Unilateral temporal infarction (#14) | Postnatal epilepsy  Bayley composite cognitive score: 82, motor: 101 (24m) |
| #11 | Recipient | post-laser TAPS | 28+0 | FLS (22) | 31+5 | 1555 (p17) | RDS | 20.5 | cPVL grade 2 (#18) | CP grade 2 |
| #12 | Recipient | post-laser TAPS | 28+0 | FLS (27) | 28+2 | 971 (p<10) | Sepsis | 21.1 | MCA stroke (#19) | Postnatal epilepsy  CP grade 2 |
| #13* | Recipient | post-laser TAPS | 23+2 | FLS (21) | 29+3 | 1009 (p<3) | Sepsis  RDS | 23.7 (2x postnatal PET) | - | Bayley composite cognitive score: 67, motor: NR (21m) |
| #14* | Recipient | post-laser TAPS | 24+2 | FLS (22) | 30+1 | 1545 (p50) | RDS | 26.4 (1x postnatal PET) | - | Postnatal epilepsy (triple WES negative)  Bayley composite cognitive score: 82, motor: 87 (24m) |
