# Supplementary material for: Brain injury in twin anemia–polycythemia sequence: prevalence, severity and long‐term neurodevelopmental outcome
Source: Ultrasound Obstet Gynecol. 2026 Mar 30;67(4):470–81. doi: 10.1002/uog.70209 (PMC13040136; doi:10.1002/uog.70209)
Supplement: Supplementary file 2 — Figure S1 Magnetic resonance imaging (MRI) of brain injury in twins with twin anemia–polycythemia sequence (TAPS). [file UOG-67-470-s002.docx]

**
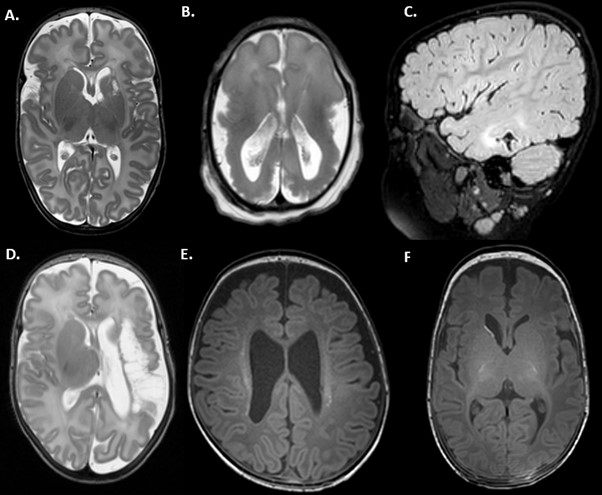
**

**Figure S1** Magnetic resonance imaging (MRI) of brain injury in twins with twin anemia–polycythemia sequence. (a) Infarction of left caudate nucleus in recipient twin with spontaneous TAPS (Case 11), born at around 30 weeks’ gestation, as shown on an axial T2-weighted image at 42 weeks post-menstrual age (PMA). (b) Significant parenchymal loss (right more than left), particularly in the parietal and occipital lobes, in the ex-donor twin following fetoscopic laser surgery at 21 weeks (Case 5), and agenesis of the corpus callosum, on an axial postmortem T2-weighted MRI. (c) Infarction in the left temporal lobe with adjacent gliosis in the recipient twin of spontaneous TAPS (Case 14), observed on a T1-weighted sagittal image at 2 years of age following development of epilepsy. (d) Main branch middle cerebral artery stroke with ipsilateral ventriculomegaly in ex-recipient after FLS (Case 19), on axial T2-weighted image at 41 weeks PMA. (e) Previously experienced intraventricular hemorrhage (IVH) and post-hemorrhagic ventricular dilation with bilateral focal punctate white matter abnormalities and diffuse white matter loss, in recipient twin with spontaneous TAPS (Case 9) on axial T1-weighted image at 48 weeks. (f) Infarct in the right caudate nucleus, in recipient with spontaneous TAPS (Case 16) on axial T1-weighted image at 44 weeks PMA.
